# Inflammatory Fibroid Gastric Polyps (Vanek’s Tumor): Two Case Reports Highlighting Epidemiological Patterns and Telocyte-Driven Neoplastic Pathogenesis and Diagnosis

**DOI:** 10.3390/reports9010002

**Published:** 2025-12-19

**Authors:** Roberto Venuto, Caterina Elisabetta Rizzo, Francesco Loddo, Giovanni Genovese, Maria Teresa Martorana, Cristina Genovese, Francesco Fedele

**Affiliations:** 1 Department of Chemical, Biological, Pharmaceutical and Environmental Sciences, University of Messina, 98166 Messina, Italy; 2 Department of Prevention, Local Health Authority of Messina, 98123 Messina, Italy; 3 Department of Prevention, Local Health Authority of Catania, 95124 Catania, Italy; caterina.rizzo93@gmail.com; 4 Department of Biomedical and Dental Sciences and Morphofunctional Imaging, University of Messina, 98123 Messina, Italy; france.loddo@gmail.com (F.L.); gigenovese@unime.it (G.G.); crigenovese@unime.it (C.G.); f.fedele1965@libero.it (F.F.); 5 Department of Human Pathology of the Adult and Evolutive Age “Gaetano Barresi”, University of Messina, 98125 Messina, Italy; teresa.martorana@studenti.unime.it

**Keywords:** vanek’s tumor, inflammatory fibroid polyp, telocytes, PDGFRA, CD34

## Abstract

**Background and Clinical Significance**: Inflammatory fibroid polyp (IFP), also known as Vanek’s tumor, is a rare, benign mesenchymal lesion of the gastrointestinal (GI) tract that frequently mimics neoplastic conditions due to its submucosal location and radiologic appearance. Although most commonly found in the gastric antrum, IFPs may occur throughout the GI tract and present with a range of symptoms, from incidental findings to obstruction or bleeding, depending on size and location. **Case Presentation**: This article presents two distinct cases of gastric IFP managed at the University Hospital of Messina: one endoscopically resected polyp in a 70-year-old man and one surgically excised infiltrative lesion in a 64-year-old woman with high-grade obstruction. Histological analysis in both cases confirmed the diagnosis of IFP, demonstrating spindle cell proliferation with eosinophilic infiltrates and a characteristic perivascular “onion-skin” pattern. Immunohistochemical staining showed strong CD34 positivity and absence of CD117 and DOG1, aiding in differentiation from gastrointestinal stromal tumors (GISTs). **Conclusions:** Recent evidence suggests a neoplastic origin for IFPs, supported by the presence of PDGFRA mutations and telocyte involvement, prompting a reevaluation of their pathogenesis. These cases underscore the diagnostic challenges posed by IFPs and highlight the importance of histological and immunohistochemical analysis in guiding appropriate treatment. While endoscopic resection is preferred for localized lesions, surgical intervention remains necessary in complex or obstructive cases. Understanding IFPs’ molecular profile and cellular origin may refine future diagnostic and therapeutic approaches.

## 1. Introduction and Clinical Significance

Inflammatory fibroid polyp (IFP), also known as Vanek’s tumor, is a rare, benign, mesenchymal lesion of the gastrointestinal (GI) tract. First described by Vanek in 1949 as a “gastric submucosal granuloma with eosinophilic infiltration” [1], IFPs are now recognized as distinct pathological entities, often mistaken for neoplastic processes due to their mass-forming nature and submucosal growth pattern [2].

Epidemiologically, IFPs account for less than 1% of all gastric polyps and a small fraction of all GI tract lesions [3]. They can occur at any age but are most frequently diagnosed in adults between the fifth and seventh decades of life, with a slight female predominance [4]. The stomach is the most common site of occurrence, particularly the antrum, followed by the small intestine (notably the ileum), colon, and, rarely, the esophagus. Lesions in the small bowel are more likely to cause obstructive symptoms, such as intussusception [5], while gastric lesions are usually larger and may present with nonspecific upper gastrointestinal symptoms or be found incidentally during endoscopy or laparotomy [6].

Despite their benign nature, the clinical presentation of IFPs is variable and highly dependent on their size and location. Small lesions are often asymptomatic, while larger polyps may cause abdominal pain, gastrointestinal bleeding, anemia, or bowel obstruction [7]. Because of their submucosal location and radiologic appearance, IFPs are often mistaken preoperatively for more aggressive lesions such as gastrointestinal stromal tumors (GISTs), leading to diagnostic uncertainty [8].

Histologically, IFPs are characterized by a proliferation of bland spindle or stellate stromal cells arranged around blood vessels in a concentric, onion-skin pattern, accompanied by a mixed inflammatory infiltrate rich in eosinophils. Immunohistochemistry typically demonstrates strong CD34 positivity and negative staining for CD117 (c-KIT), DOG1, S-100, and desmin, distinguishing them from other GI mesenchymal tumors [9].

In recent years, growing molecular evidence has challenged the long-held belief that IFPs are purely reactive lesions. Several studies have identified activating PDGFRA mutations in a substantial proportion of cases, supporting a neoplastic origin and suggesting a pathogenetic pathway that partially overlaps with that of GISTs [10]. Moreover, emerging research on telocytes—specialized stromal cells that co-express CD34 and PDGFR—has highlighted their potential role as the true cells of origin of IFPs [11]. These findings have intensified the debate regarding the neoplastic versus reactive nature of IFPs and underscore the importance of integrating molecular and cellular insights into their characterization.

In this article, we describe two clinically and anatomically distinct cases of inflammatory fibroid polyps reported in the University Hospital “G. Martino” of Messina, emphasizing the diagnostic process, therapeutic intervention, and histopathological features. Both cases were identified through a retrospective review of all submucosal gastric lesions evaluated at our institution over a five-year period. During this interval, only two cases fulfilled the histological, immunohistochemical, and molecular diagnostic criteria for inflammatory fibroid polyp, underscoring the exceptional rarity of this entity in routine clinical practice. The selected cases represent the full clinical spectrum observed, ranging from an incidentally detected, polypoid lesion amenable to endoscopic resection to a large, infiltrative mass requiring surgical management.

No statistical analyses were conducted given the descriptive nature and limited sample size of this case-based study.

## 2. Case Presentations

### 2.1. Case Report 1

A 70-year-old male patient was found to have sideropenic anemia and leukothrombocytosis during routine hematochemical testing. This prompted further investigations, including a bone marrow biopsy, which revealed a well-cellular hematopoietic marrow with no definitive morphological signs of myeloproliferative neoplasia. Peripheral blood flow cytometry did not reveal significant abnormalities, and a JAK2 mutation test was negative. The patient also underwent esophagogastroduodenoscopy (EGD).

The patient’s medical history included type 2 diabetes, ischemic heart disease, coronary angioplasty with drug-eluting stent placement in the circumflex artery, chronic hemispheric hemorrhage on the left side, bipolar disorder, dyslipidemia, hepatic steatosis, and chronic obstructive pulmonary disease (COPD).

EGD revealed a nodular-appearing gastric wall, warranting biopsy for histological evaluation and *Helicobacter pylori* testing. Additionally, a polypoid, pedunculated lesion approximately 3 cm in diameter was observed in the pre-pyloric antrum, surrounded by hyperemic mucosa. Biopsy of the gastric body showed no neoplastic changes, and *H. pylori* was not detected.

Endoscopic ultrasound (EUS) characterized the lesion as a homogenous, hypoechoic mass within the second and third sonographic layers. The lesion was removed en bloc using endoscopic mucosal resection (EMR) with the “inject-and-cut” technique, including endoscopic hemostasis via epinephrine (1:20,000 in normal saline). No immediate post-procedural complications occurred, and an endoclip was placed at the polyp base to ensure hemostasis. Follow-up endoscopy showed no signs of local recurrence.

**Figure 1:**
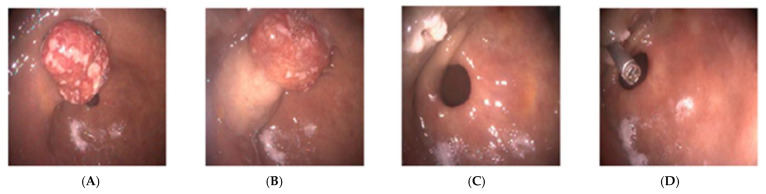
Endoscopic examination of the lesion (**A**) mushroom-shaped polypoid lesion; (**B**) epinephrine injections; (**C**) polyp base post-resection; (**D**) endoclip placement.

Endoscopic images included: a 3.3 × 3 × 2 cm multi-lobed, mushroom-shaped polypoid lesion (Figure 1).

**Figure 2:**
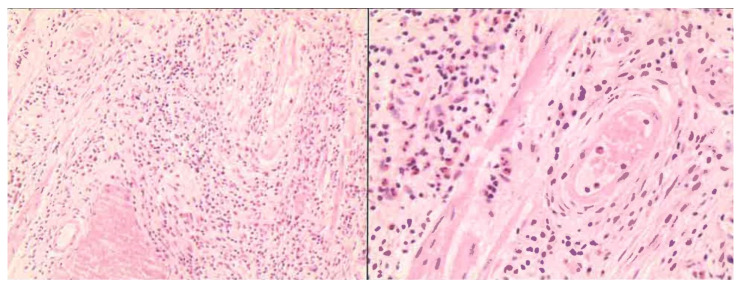
Microscopy evaluation of the lesion (**left**: 10×; **right**: 40×).

Microscopic examination revealed a partially ulcerated polypoid lesion with submucosal growth, composed of spindle-shaped, dendritic, fibroblast–myofibroblast, and epithelioid mesenchymal stromal cells. These exhibited finely granular chromatin, small nucleoli, scant cytoplasm, rare mitoses, and a concentric, “onion-bulb” growth pattern around small and medium-sized vessels. The stroma was myxoid and inflamed, with significant lymphocytic and eosinophilic infiltrate, edema, and collagenization (Figure 2).

**Figure 3:**
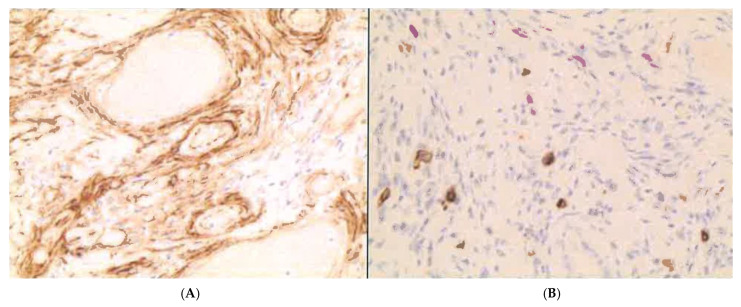
Immunoreactivity for CD34 (**A**) (20×) and CD117 (c-kit) (**B**) (40×).

Immunohistochemistry showed strong positivity for Vimentin, CD34, and smooth muscle actin, with negative results for S-100, CD117 (c-Kit) (Figure 3). The proliferation index was low, as indicated by MIB-1 (Ki-67) staining.

Overall, the histological, immunohistochemical, and molecular findings support a diagnosis of benign inflammatory fibroid polyp (Vanek’s tumor) [12].

### 2.2. Case Report 2

We report the case of a 64-year-old woman who presented with recurrent episodes of abdominal colicky pain localized to the epigastric and right hypochondriac regions, typically worsening immediately after meals. The pain was temporarily relieved with analgesic and antispasmodic medications.

Her medical history was notable for arterial hypertension and severe obesity (BMI 46.8 kg/m^2^). Laboratory tests revealed microcytic anemia (Hb 12.1 g/dL), normal lactate dehydrogenase (LDH) levels, mild hypergammaglobulinemia, and newly diagnosed diabetes mellitus with an HbA1c of 9.3%.

Tumor markers were within normal limits. Upper endoscopy (OGDS) showed deformation of the antro-pyloric region, suggestive of extrinsic compression, without mucosal lesions, with the impossibility of performing an endoscopic biopsy. Abdominal CT scan revealed a multiloculated mass measuring 7 × 5 cm between the body and antrum of the stomach along the greater curvature, primarily on the anterior wall. The lesion appeared to infiltrate the gastric wall, causing significant luminal narrowing. It exhibited peripheral enhancement, internal hypodensity indicative of cystic or necrotic degeneration, and developed mainly along the serosal surface. Enlarged omental lymph nodes (up to 14 mm) and severe hepatic steatosis were also noted.

Differential diagnoses included GIST or lymphoma, though other neoplasms could not be excluded.

During hospitalization, the patient developed a high-grade intestinal obstruction. An attempt at endoscopic ultrasonography (EUS) failed due to the inability to distend the deformed antro-pyloric region and visualize gastric wall layers.

Given the acute presentation, the patient underwent exploratory laparoscopy with intraoperative biopsy of perigastric tissue in the antral region. A subtotal gastrectomy with lymphadenectomy, Roux-en-Y reconstruction, and total omentectomy was performed.

Frozen section analysis revealed lymphoid tissue infiltrating adjacent adipose tissue in both the perigastric area and the falciform ligament.

**Figure 4:**
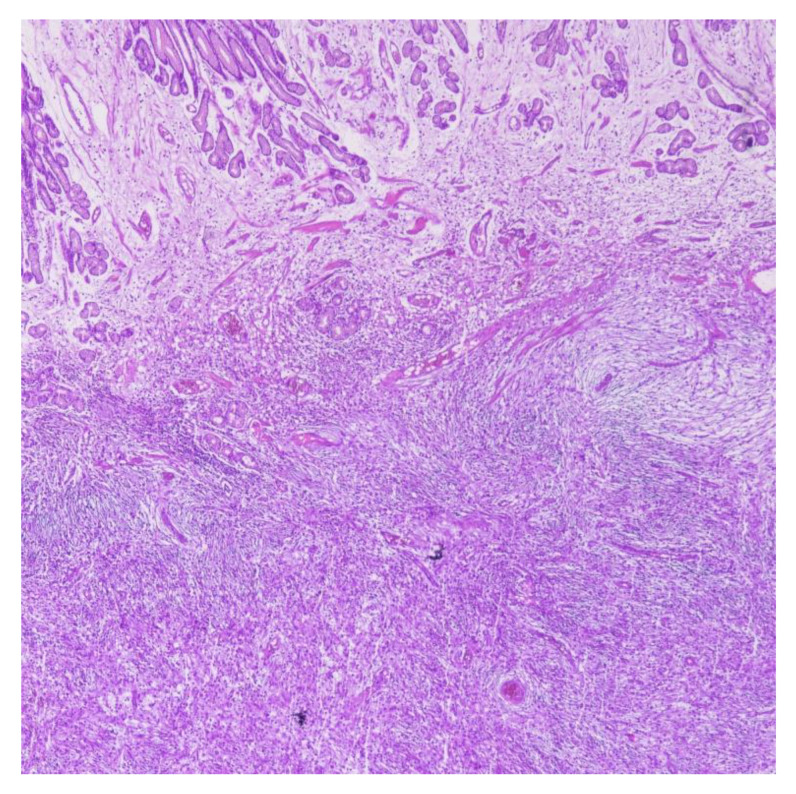
Microscopy evaluation of the lesion (4×).

Final histological examination confirmed chronic inflammatory infiltration of the falciform ligament, gastric wall, and greater omentum. A 2.5 × 7 cm antral tumor was identified in the anterior gastric wall, displaying mononuclear, spindle-shaped cells arranged in whorls with diffuse eosinophilic infiltration, determining polypoid intramural protrusion, consistent with an inflammatory fibroid polyp (IFP), with GIST considered in the differential diagnosis (Figure 4).

**Figure 5:**
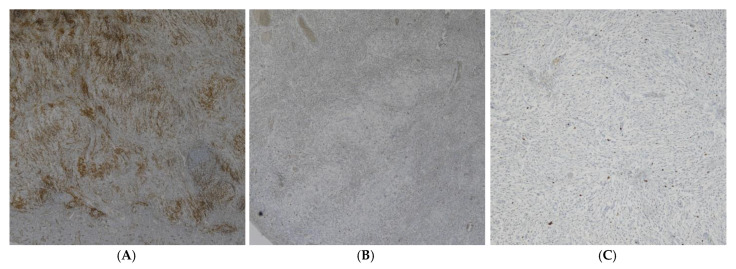
Immunoreactivity (10×) for CD34 (**A**), CD117 (c-kit) (**B**) and MIB-1 (**C**).

Immunohistochemistry showed strong positivity for CD34 and negativity for CK AE1/AE3, Desmin, AML, S-100, and CD20 (Figure 5). These findings confirmed the diagnosis of Vanek’s tumor.

The patient was discharged two weeks postoperatively without symptoms [11]. At 14-month follow-up, abdominal CT showed postoperative changes consistent with partial gastrectomy. No free fluid was present in the abdominal or pelvic cavities. Importantly, there was no evidence of recurrence of the inflammatory fibroid polyp (Vanek’s tumor), confirming the success of surgical treatment [13].

## 3. Discussion

Numerous terms have been proposed to describe IFP, including eosinophilic granuloma, granuloblastoma, gastric fibroma with eosinophilic infiltration, granuloma with eosinophils, hemangiopericytoma, inflammatory fibroid tumor, and inflammatory pseudotumor [14,15]. The wide variety of these names highlights the difficulty to understand the precise mechanisms behind IFP development and the different theories about its origin [16].

Historically, IFP was considered to be a reactive inflammatory condition. It was believed that IFP could be linked to factors such as *Helicobacter pylori* infection [17,18] or type A gastritis. It has been suggested that local infections (in fact, some reported IFP cases are associated with microorganisms such as Cytomegalovirus [19] and *Actinomyces israelii* [20]), allergic reactions, autoimmune responses, or an exaggerated host response to an unidentified trigger may all contribute to the development of IFP [21]. Some researchers suggest that IFP might result from chronic irritation and inflammation or from an exaggerated bodily response to intestinal trauma, or even as a localized form of eosinophilic gastroenteritis, given its prominent eosinophilic infiltration. The common occurrence of these lesions in the stomach, where strong contractions and coarse food may cause local irritation, supports a traumatic origin.

Additionally, a few cases have been reported linking IFP with Crohn’s disease [22].

Due to the unclear cause of IFP, the possibility that this typically benign tumor may have malignant potential remains a topic of ongoing debate. Instances of familial occurrence or the simultaneous development of IFP and other gastrointestinal cancers indicate that some malignant potential might exist, though it is rare [23].

From an immunohistochemical perspective, IFPs are characterized by spindle-shaped cells that test positive for CD34, PDGFRA, and vimentin, but negative for CD117, DOG1, and S100 [24].

The differential diagnosis of IFPs includes various spindle cell lesions with inflammatory infiltrates. Their consistent expression of cyclin D1 and fascin suggests possible dendritic cell differentiation, but the hallmark is CD34 positivity in small lesions, which may decrease as they enlarge. This, along with molecular features, can raise concern for GISTs; however, IFPs differ morphologically and lack CD117 (c-Kit) expression [25].

Larger IFPs may mimic sarcomas, but their bland cytology and inflammatory background argue against malignancy. They may also resemble nodular fasciitis, which is CD34-negative and strongly actin-positive [26]. Solitary fibrous tumors share CD34 expression but are rare in the GI tract and lack inflammation. Notably, up to 8% of IFPs occur alongside adenomas or carcinomas [23].

To aid diagnostic clarity, Table 1 summarizes the main differential diagnoses of gastric spindle cell and mesenchymal tumors, highlighting histological and immunohistochemical features that distinguish IFPs from other entities.

In 55–74% of IFP cases the presence of oncogenic PDGFRA mutations, affecting specifically exons 12, 14 and 18, is observed, as in other stromal tumors (GIST), suggesting that these lesions may arise due to the activation of PDGFRA [27].

Building on this evidence, recent molecular studies have clarified the distribution and implications of these mutations. Exon 12 mutations—often deletions or deletion–insertions—are more common in small-intestinal IFPs, whereas gastric lesions frequently harbor exon 14 or exon 18 mutations, including the characteristic D842V substitution. Importantly, these same PDGFRA alterations are found in up to 40% of GISTs, highlighting a shared molecular pathway despite the clearly benign behavior of IFPs. Unlike PDGFRA-mutant GISTs, which typically exhibit epithelioid cytology, IFPs maintain a spindle-cell phenotype, underscoring their distinct histopathological identity.

The link between PDGFRA activity and eosinophilic inflammation adds another dimension to IFP pathogenesis. Although the FIP1L1–PDGFRA fusion—well established in idiopathic hypereosinophilic syndrome—has not been identified in IFPs, it illustrates how PDGFRA dysregulation can drive marked eosinophilic proliferation. Additionally, PDGFRA rearrangements have been increasingly described in myeloid neoplasms with eosinophilia, further supporting a mechanistic relationship between this signaling pathway and the eosinophil-rich stroma characteristic of IFPs. More recently, exon 15 BRAF mutations have been reported in PDGFRA-wild-type IFPs, expanding the spectrum of known molecular alterations and reinforcing the notion that these lesions represent true benign neoplasms rather than reactive proliferations [10].

Recently, some Authors identified the spindle cells displayed by this lesion in telocytes (TCs) [28]: they are mesenchymal, stromal cells, formerly known as interstitial Cajal-like cells, expressed in a variety of tissues and organs, morphologically characterized by a nucleated body with long and thin prolongations, called telopodes [29,30].

In the gastrointestinal tract, telocytes uniquely co-express CD34 and PDGFR while lacking c-Kit expression, unlike in other organs. These distinct features allow for their reliable differentiation from interstitial cells of Cajal, particularly through PDGFR-based immunohistochemistry [31].

Beyond immunophenotypic similarity, telocytes are now considered central to IFP pathogenesis. Their extensive intercellular communication network, regulatory role in stromal architecture, and demonstrated ability to respond to PDGFRA-driven signaling provide a mechanistic framework in which telocytes may undergo PDGFRA-mediated neoplastic transformation. This dual involvement of telocytes and PDGFRA mutations helps reconcile the paradox of IFPs: a lesion that behaves clinically as a benign process yet exhibits clear molecular features of neoplasia. Telocytes’ intrinsic stromal regulatory functions may also explain the characteristic onion-skin arrangement and eosinophil-rich microenvironment, reflecting an aberrant—yet self-limited—stromal remodeling response.

These cells form an intricate communication network among various stromal and epithelial cell types [32]; furthermore, they should be the androgen receptor-positive cells found on the peripheral part of onion skin-like formations and corresponding with Ki67-positive cell distribution, described by some authors, suggesting a hormonal influence in the development of this tumor. Additionally, the development of Vanek’s tumor exhibits similarities to the progression of androgen-sensitive prostate cancer, which is influenced by PDGF [33].

Ricci et al. proposed an additional term for defining this lesion, “telocytoma”, as it clearly synthetizes the origin cell (telocyte) and the neoplastic pathogenesis [34].

Although traditionally classified as a benign reactive lesion, evolving molecular and immunophenotypic data increasingly support a neoplastic origin for IFPs. This has important clinical implications for diagnosis, management, and surveillance. The detection of activating mutations in the PDGFRA gene, found in a significant proportion of IFPs, argues against a purely reactive etiology and instead aligns them closer to other neoplastic stromal lesions such as GISTs, though with a distinctly benign biological behavior [35]. The clinical variability between the two cases presented in this report underscores the diverse manifestations of IFPs. Case 1, involving a pedunculated lesion in the gastric antrum, was managed successfully through minimally invasive endoscopic mucosal resection, consistent with the typical treatment of small, localized IFPs. In contrast, Case 2 involved a larger, infiltrative mass mimicking a malignant neoplasm and causing high-grade obstruction, ultimately necessitating surgical resection. This demonstrates the importance of including IFP in the differential diagnosis of submucosal or infiltrative GI masses, particularly when imaging suggests potential malignancy.

While IFPs rarely recur following complete resection, their preoperative misidentification as GISTs or other neoplastic lesions often leads to overtreatment. Accurate diagnosis therefore depends heavily on histopathological assessment, particularly the identification of hallmark concentric perivascular fibrosis and eosinophil-rich inflammatory stroma. Immunohistochemical staining remains a critical diagnostic adjunct; IFPs show diffuse CD34 positivity and lack staining for CD117 and DOG1, which helps distinguish them from GISTs. Importantly, while CD34 is not exclusive to IFPs, its combination with the lesion’s architectural and inflammatory features is diagnostic in the appropriate clinical context.

The association of IFPs with other inflammatory or immune-mediated conditions, as well as the presence of telocytes as a putative origin cell, opens new research avenues. Telocytes are increasingly recognized as critical regulators of local tissue architecture and intercellular signaling, and their proposed role in IFP pathogenesis, especially in tumors harboring PDGFRA mutations, may explain the neoplastic but non-aggressive behavior of these lesions [29]. Furthermore, the possible hormonal influence, indicated by androgen receptor expression in some cases, merits deeper investigation, especially given reports of sex-related incidence patterns and similarities with hormone-sensitive tumors such as prostate cancer.

From a clinical management perspective, awareness of PDGFRA-driven pathogenesis may help refine therapeutic decision-making, especially in large or infiltrative lesions where malignancy is suspected preoperatively. Although targeted therapies are not indicated for IFPs, molecular confirmation of PDGFRA mutations may reinforce conservative management following complete resection and reduce the need for aggressive surgical approaches. Moreover, the emerging recognition of telocytes as the putative cells of origin provides a biological explanation for the unique stromal organization and eosinophil-rich microenvironment of IFPs, bridging the gap between their neoplastic nature and indolent clinical behavior.

These molecular insights may also guide future diagnostic protocols, encouraging the integration of molecular testing in selected cases and supporting a more personalized, evidence-based approach to the management of gastrointestinal mesenchymal lesions.

In recent years, artificial intelligence (AI) has emerged as a powerful tool in gastrointestinal endoscopy, radiology, and histopathology, with significant implications for the diagnosis of gastric subepithelial lesions. Machine learning and deep learning algorithms have demonstrated high accuracy in detecting and characterizing gastric lesions during gastroscopy, particularly when integrated with narrow-band imaging (NBI) and magnification endoscopy. These technologies may be especially valuable in differentiating inflammatory fibroid polyps from other mesenchymal tumors such as gastrointestinal stromal tumors, which often present with overlapping endoscopic and radiologic features.

AI-assisted image analysis can enhance real-time lesion recognition, margin delineation, and risk stratification during endoscopy, potentially improving early detection and guiding decisions regarding endoscopic versus surgical management. Furthermore, AI-based histopathological analysis may support pathologists by identifying subtle architectural patterns—such as the characteristic perivascular onion-skin arrangement—and immunophenotypic signatures associated with IFPs. Radiological applications of AI may also contribute to more accurate assessment of lesion depth, growth pattern, and infiltrative behavior, reducing diagnostic uncertainty in complex cases.

As AI technologies continue to evolve, their integration into clinical workflows could enable more precise, personalized diagnostic pathways for patients with rare gastric lesions such as IFPs, ultimately reducing overtreatment and improving clinical outcomes [36,37,38].

Future research should focus on expanding molecular profiling of IFPs in larger multicenter cohorts to better define genotype–phenotype correlations and to clarify whether specific PDGFRA or BRAF mutations are associated with lesion size, anatomical location, or clinical presentation. Further investigation into telocyte biology may also elucidate the mechanisms underlying stromal remodeling, eosinophil recruitment, and the self-limited growth of these tumors. Additionally, prospective studies evaluating the role of AI-assisted endoscopy and digital pathology in the diagnosis of rare subepithelial lesions could help standardize diagnostic algorithms and reduce interobserver variability.

From a therapeutic perspective, endoscopic resection remains the preferred treatment for accessible, well-circumscribed IFPs. According to Dias et al., endoscopic submucosal dissection (ESD) appears to be a safe and effective technique for removing gastric inflammatory fibroid polyps that manifest as large subepithelial lesions, provided it is conducted by skilled endoscopists following a thorough evaluation with endoscopic ultrasound, also for polyps of more than 20 mm in size [4].

Surgical resection is reserved for acute presentations, larger lesions, those with obstructive complications, or those in which malignancy cannot be excluded preoperatively. Long-term prognosis is excellent, with extremely rare reports of recurrence or malignant transformation. However, continued reporting of atypical presentations and larger case series will be necessary to more effectively define the complete spectrum of this lesion’s clinical behavior.

## 4. Conclusions

Inflammatory fibroid polyps are rare benign gastric lesions that often pose significant diagnostic challenges due to their clinical and radiologic resemblance to malignant tumors. Recent molecular discoveries, particularly the identification of activating PDGFRA mutations and the proposed telocyte origin, support their classification as true benign neoplasms rather than reactive lesions.

Accurate diagnosis relies on the integration of histological architecture, immunohistochemical profiling, and molecular data, which together allow for appropriate therapeutic planning and help avoid unnecessary aggressive interventions. Endoscopic resection remains the treatment of choice for localized lesions, while surgery is reserved for complex or obstructive cases. Continued investigation into molecular mechanisms and emerging diagnostic technologies, including artificial intelligence, may further refine personalized management strategies for this rare entity.

Finally, broader public-health practices—such as promoting awareness of gastrointestinal warning signs and encouraging timely medical consultation—may also help reduce delays in diagnosis and improve overall outcomes [39,40].

## Figures and Tables

**Table 1:** Differential diagnosis of gastric tumors with key distinguishing characteristics.

Lesion/Tumor Type	Histological Features	Immunohistochemistry Profile	Clinical Notes/ Distinguishing Points
Inflammatory Fibroid Polyp (IFP/Vanek’s tumor)	Bland spindle/stellate cells in onion-skin perivascular pattern; eosinophil-rich stroma	CD34+, PDGFRA+; CD117–, DOG1–, S100–, Desmin–	Benign, submucosal; may mimic GIST; may rarely cause obstruction or bleeding; PDGFRA mutations common
Gastrointestinal stromal tumor (GIST)	Spindle or epithelioid cells; variable cellularity	CD117 (c-KIT)+, DOG1+, often CD34+; PDGFRA mutations in subset	Malignant potential; requires risk stratification (size, mitotic index); responds to tyrosine kinase inhibitors
Leiomyoma/leiomyosarcoma	Fascicles of spindle cells with cigar-shaped nuclei	SMA+, Desmin+, Caldesmon+; CD117–, DOG1–, CD34–	Leiomyoma benign; leiomyosarcoma malignant; smooth muscle origin
Schwannoma	Spindle cells with palisading nuclei; peripheral lymphoid cuff	S100+, GFAP+; CD34–, CD117–, DOG1–	Benign, slow growing; neural crest origin; rare in stomach
Solitary fibrous tumor	Pattern-less architecture; collagen bands; staghorn vessels	CD34+, STAT6 nuclear+; CD117–, DOG1–	Rare in GI tract; usually benign but can recur; occasional malignant behavior
Nodular fasciitis	Myxoid stroma; tissue culture-like spindle cells; rapid growth	SMA+, Actin+; CD34–, CD117–	Reactive lesion; self-limited; may be misdiagnosed as sarcoma
Inflammatory myofibroblastic tumor (IMT)	Myofibroblastic spindle cells with inflammatory infiltrate	ALK+, SMA+, Desmin+; CD34 variable	Intermediate biologic potential; may recur; rare metastasis; uncommon in stomach
Lymphoma (gastric involvement)	Diffuse sheets of atypical lymphoid cells	CD20+ (B-cell), CD3+ (T-cell) depending on subtype	Malignant; systemic disease; may mimic submucosal mass; B-cell most common (DLBCL, MALT)

## Data Availability

The original data presented in the study are included in the article, further inquiries can be directed to the corresponding author.
